# Gyro Drift Correction for An Indirect Kalman Filter Based Sensor Fusion Driver

**DOI:** 10.3390/s16060864

**Published:** 2016-06-11

**Authors:** Chan-Gun Lee, Nhu-Ngoc Dao, Seonmin Jang, Deokhwan Kim, Yonghun Kim, Sungrae Cho

**Affiliations:** 1 School of Computer Science and Engineering, Chung-Ang University, Seoul 156-756, Korea; cglee@cau.ac.kr (C.-G.L.); dnngoc@uclab.re.kr (N.-N.D.); smjang@uclab.re.kr (S.J.); yhkim@uclab.re.kr (Y.K.); 2 Department of Vehicle Components, LG Electronics, Seoul 073-36, Korea; gimdeokhwan@gmail.com

**Keywords:** sensor fusion, indirect Kalman filter, accuracy improvement, gyro drift correction

## Abstract

Sensor fusion techniques have made a significant contribution to the success of the recently emerging mobile applications era because a variety of mobile applications operate based on multi-sensing information from the surrounding environment, such as navigation systems, fitness trackers, interactive virtual reality games, *etc*. For these applications, the accuracy of sensing information plays an important role to improve the user experience (UX) quality, especially with gyroscopes and accelerometers. Therefore, in this paper, we proposed a novel mechanism to resolve the gyro drift problem, which negatively affects the accuracy of orientation computations in the indirect Kalman filter based sensor fusion. Our mechanism focuses on addressing the issues of external feedback loops and non-gyro error elements contained in the state vectors of an indirect Kalman filter. Moreover, the mechanism is implemented in the device-driver layer, providing lower process latency and transparency capabilities for the upper applications. These advances are relevant to millions of legacy applications since utilizing our mechanism does not require the existing applications to be re-programmed. The experimental results show that the root mean square errors (RMSE) before and after applying our mechanism are significantly reduced from $6.3\times 10^{-1}$ to $5.3\times 10^{-7}$, respectively.

## 1. Introduction

Currently, the mobile applications era is looking forwards to the next generation where a virtual personal assistant (VPA) performs the central unified framework, which integrates separate applications to provide humans with context-aware personalized information and facilities [1]. Cooperating with various advanced technologies, sensor fusion plays an important role in improving the accuracy of multi-sensing information from the surrounding environment, which is a vital input data for a VPA to correctly respond to the user’s requests or for the user experience features for interactive relaxation applications, *etc.* [2]. By utilizing the Internet of Things (IoT) infrastructure, we are able to interconnect with many kinds of sensors. However, within the human activities of daily living (HADL) focus, accelerometer and gyroscope sensors are the most popular objects, which have already been installed in billions of smartphones nowadays. Moreover, this progress is forecast to continue for at least the next decade [3].

However, due to the rapid upgrading of smartphones, mobile applications are facing difficulties in that the code requires modification to adapt to additional hardware components in new devices. This problem negatively affects billions of existing smartphones in the world and millions of applications in the market stores [4]. For instance, the pedometer sensor was introduced in the Google Nexus 5 (Google, San Francisco, CA, USA), Samsung Galaxy S5 (Samsung, Seoul, Korea), and Apple iPhone 5S (Apple, Cupertino, CA, USA) generations. Therefore, step-count information provided by the pedometer cannot be taken into account without modifications of their code. Moreover, even if the developers try to modify and re-compile the code to utilize the sensor fusion technique for upgrading their applications, another problem arises because the applications separately apply sensor fusion using their own approach. This not only burdens the smartphone’s performance, but also creates more latency in the processing time [5].

Although there are existing solutions which implemented sensor fusion in kernel space as well as user space, typical implementations of Kalman-based sensor fusion require external feedback loops between the components that are requesting and providing the services. Some solutions are installed at firmware level according to different underlying hardware; as a consequence, they are inflexible and inconvenient to be updated. On the other hand, the solutions implemented in the application level often exhibit lower performance compared to kernel space solutions in many operating systems; even worse, all deployed applications have to be re-programmed when the underlying hardware is changed or updated. This is critical because there are billions of deployed applications nowadays.

Attempts to address these aforementioned problems have achieved significant success by using some interesting approaches [6]. The existing solutions have applied a variety of theoretical frameworks to build effective fusion algorithms for imperfect data (see Section 2 for more detail). However, almost all approaches concentrate only on improving the accuracy and fusion calculating performance without any consideration of implementation position or facilitation for developers, which is very important in the emerging mobile applications era [7].

From this point of view, we have proposed a new solution that implements the well-known Kalman filter for sensor fusion in the device-driver layer. The experimental results show that our solution provides greater convenience, benefiting the developer by making the applications independent of the underlying sensor hardware upgrade. A part of our work has been presented at the third International workshop on smartphone applications and services, 2011 [8]. Based on these achievements, we enhance the accuracy and processing performance by using a quaternion based indirect Kalman filter and develop additional feedback components to correct the gyro drift problem. The gyroscope and accelerometer values are pre-calculated before reaching the corresponding applications.

Our main contributions in this paper are summarized as follows:

- We have proposed a new software architecture for sensor fusion driver utilizing the quaternion based indirect Kalman filter in conjunction with additional feedback components for gyro drift correction. These components do not only handle the external feedback loop issue between the device driver and applications, but also cancel the non-gyro signal in the measured state vector.
- The developed sensor fusion driver abstracts underlying sensor hardware to provide an unified framework for mobile applications. The multi-sensing information is facilitated without any requirement of re-programming or modification in the existing applications. It supports backward compatibility for the legacy applications as well.
- The implementation in the device driver layer provides greater performance up to 10 times from 538 to 5,347 samples per second, and lower latency in calculation time from 1.8579 ns to 0.18702 ns. The duplication of sensor fusion process among applications is completely addressed.

The remainders of this paper are organized as follows. Section 2 classifies the related work of different approaches into corresponding categories. Section 3 defines the problem statement and our approach to resolve it. The proposed solution is described in Section 4. Section 5 shows the experimental results and discussion. Conclusions are drawn in Section 6.

## 2. Related Work

As mentioned above, in the scope of this paper, we are concerned about sensor fusion problems that happen in smartphone environments where VPA applications are closely installed. Therefore, the survey of related work has concentrated on existing solutions for imperfect data from multi-sensing information. We classify the related works into two groups by systematical designs, including centralized calculation and local calculation. In both groups, the existing solutions are also divided into theoretical approaches: probabilistic, statistic, and artificial intelligence.

### 2.1. Systematical Designs

The partial-centralized and fully centralized calculation approaches are typical models that were introduced in past decades. Based on the network architecture, within the clustering and tree-topology models, the multi-sensing information might be roughly pre-filtered at the clustering head nodes or the *i-th* level root nodes before reaching the centralized processing server [5,9,10]. The centralized calculation approach provides the outstanding advantages of high performance, complex mathematical solutions, social information sharing, unified IoT framework, *etc.* [11,12,13]. However, in some special cases, e.g., VPA applications, it reveals limitations of response latency, assisted-information localization, and personal data privacy.

In contrast, the local calculation approach is able to compensate for the above limitations. However, due to its natural disadvantages, nowadays, local calculation models only play a supplemental role in the centralized solution as a local agents [10,11,14]. The combination of two approaches almost satisfies the HADL applications’ requirements.

### 2.2. Theoretical Approaches

Based on theoretical approaches, our taxonomy classifies the existing related work into three categories, including a probabilistic method, statistical method, and artificial intelligence application method.

Within the probabilistic methods, the probability distribution functions of multi-sensing data are fused together using Bayesian analysis. The rationale of this method is that since the sensing information from related sources has properties of concrete probability distributions, combinations of this information will be able to better correct the imperfect data [4,6]. This approach is only effective when the related data has well-known distributions and the environment’s conditions are stable. One exceptional case of Bayesian analysis is the well-known Kalman filter and its variations. The orientation correction and error reduction of the Kalman filter based sensor fusion technique were significantly improved by a variety of research such as the effective solution proposed by Van Der Merwe in [15]. Nowadays, the Kalman filter is one of the most popular fusion methods, which is implemented in various sensing domains due to its simplicity and acceptable accuracy [16,17].

Applying the statistical method, the evidence of sensing data from multi sensors are collected and analyzed during the processing time [4]. Using the Dempster-Shafer evidence accumulation rule, the probability mass function is fused to recognize the properties of the data and predict the trend of the next sensing value. The Depster-Shafer framework allows different information levels from multi resources to be combined, depending on its weight factor [5,18]. It makes more flexible to reproduce the desired sensing data. Besides that, the context-aware, semantic information matching [19], and fuzzy reasoning methods [20,21] are also utilized to adjust the output data as required.

Recently, the artificial intelligence method is becoming popular in the information fusion field. Almost proposed solutions are centralized models where the multi-sensing data is gathered into a logical central processing server. The complex algorithms are performed using neural networks [22,23] and machine learning techniques [24] for analyzing and combining sensing data to obtain the desired information. Moreover, the emerging cloud-fog computing infrastructure also provides better performance for artificial intelligence algorithms.

## 3. Preliminaries

### 3.1. Problem Statement

**Figure 1:**
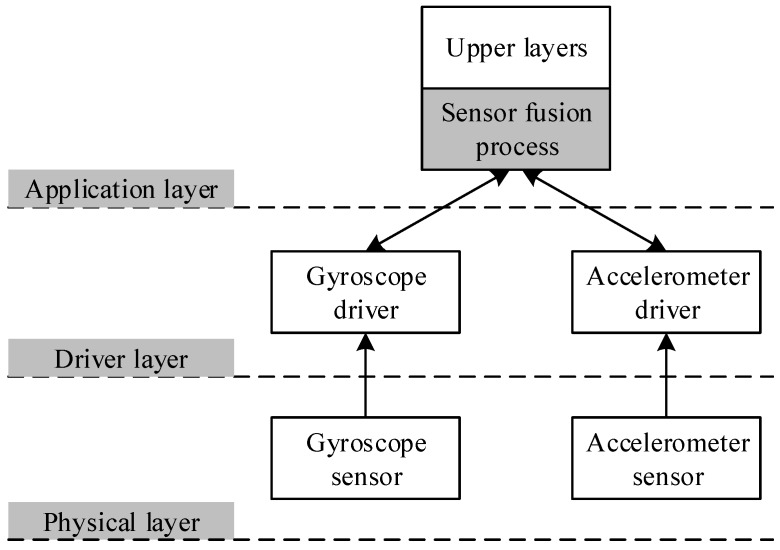
Sensor fusion processes in the legacy methods.

Following the above survey, since the outstanding properties of the Kalman filter and its variations are simplicity in implementation and acceptable accuracy for almost all HADL applications, nowadays, these filters are the most popular sensor fusion methods and are widely integrated into personal devices such as smartphones, smartwatchs, and wearable devices. The position at which the Kalman filters are implemented is also an interesting topic inspiring researchers to pay attention. The dominant strategies focus on the application layer (*i.e.*, internal process of stand alone applications) and remote processing (*i.e.*, cloud and fog computing). The general architecture of legacy sensor fusion methods is described in Figure 1. Whenever the application requests sensing information, it must simultaneously contact multi sensors through separate corresponding drivers. The overhead in request and response operations rapidly increases, proportionally to the number of related sensors. Besides that, the fusion process is calculated in the application layer, which might cause significant latency and cannot be acceptable by real-time applications.

The strategy to implement sensor fusion in the lower layer is even more challenging due to the difference in protocols and interface standards among sensor components. For instance, within our proposal, we intend to apply the indirect Kalman filter at the device driver level to obtain better performance and lower latency and overhead, we are facing some challenges as follows:

- *External feedback loop:* There is a feedback loop between the modules of the filter. If the indirect Kalman filter were directly applied, it would result in a feedback loop signal between the applications and the device driver, which is not a desirable configuration. When the Kalman filter is implemented in the device driver level, it would use Euler angle kinematics in calculation, which is not linear. The orientation value is transformed to gyro value before coming out of the Kalman filter, then it has to be converted to orientation value again inside the applications. The repeated transformations may cause error accumulations because Euler angle kinematics is not linear. Note that the feedback loop is outside of the Kalman filter.
- *Non-gyro signal in the measured state vector:* Within the original indirect Kalman filter, a non-gyro signal always appears in the state vector after measurement. It is not hard to resolve this problem in the desired applications, but it wastes more time.

### 3.2. Our Approach

In the emerging mobile application era, especially focusing on the VPA framework, quick and exact response capability is very important. Moreover, since the applications are implemented in mobile and wearable devices, which have limited hardware resources and battery, the processing overhead must also be considered. To satisfy these requirements, we utilized a driver-based approach. Our proposed sensor fusion architecture was developed at the driver layer to provide a unified framework where the measured sensing data is collected and processed before reaching the upper applications.

Due to its popularity and simplicity, the indirect Kalman filter is integrated into the framework to perform information fusing functions. In order to address the external feedback loop and non-gyro signal in the measured state vector issues, we apply the quaternion based method on the indirect Kalman filter incorporating additional modules to return the required gyro and accelerometer values at the corresponding output interfaces.

## 4. Proposed Solution

### 4.1. Sensor Fusion Driver Architecture

**Figure 2:**
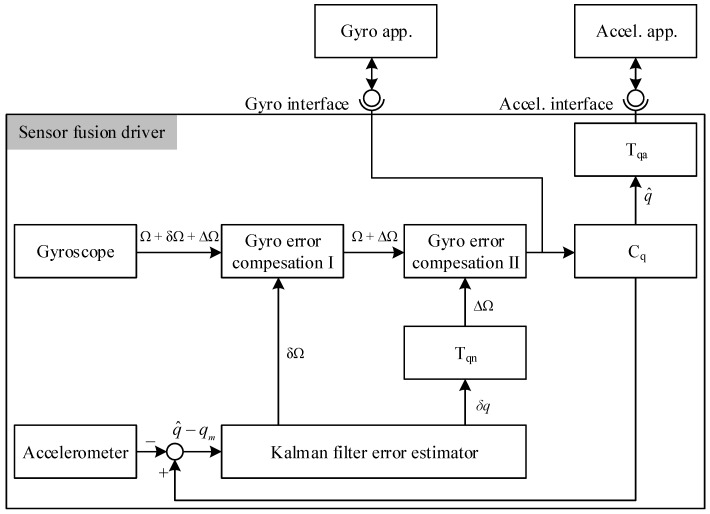
The proposed sensor fusion driver architecture.

Figure 2 shows our proposed sensor fusion driver architecture, which consists of a quaternion based indirect Kalman filter and three additional modules named $T_{qn}$, $T_{qa}$, and $C_{q}$. The gyro interface and accelerometer interface separately serve applications using their expected gyro and accelerometer information, respectively.

In the figure, Ω, δΩ, and ΔΩ represent the angular velocity, gyro bias error, and gyro drift rate noise, respectively, and $q_{m}$ is the measured value received from the accelerometer sensor. The quaternion value $\hat{q}$ is defined as

(1) $$\hat{q}=q_{4}+q_{1}i+q_{2}j+q_{3}k$$

where *i*, *j*, and *k* are hyper-imaginary numbers, $q_{4}$ represents the real part of the quaternion, which is equal to

(2) $$q_{4}=\cos\left(\theta /2\right)$$

where *θ* is the angle of rotation [25].

Denote the measured value received from the gyro sensor by $w_{m}$. The noise model of the gyro sensor is derived as follows

(3) $$w_{m}=\Omega +\delta \Omega +\Delta \Omega$$

The purposes of gyro error compensation modules I and II is to remove the δΩ and ΔΩ signals, respectively. The error state vector $\hat{x}$ of the indirect Kalman filter can be expressed as

(4) $$\hat{x}=\left[\begin{array}{cccccc} \delta q_{1} & \delta q_{2} & \delta q_{3} & \delta q_{x} & \delta q_{y} & \delta q_{z} \end{array}\right]$$

where $\delta q=\left[\begin{array}{ccc} \delta q_{1} & \delta q_{2} & \delta q_{3} \end{array}\right]$.

### 4.2. Gyro Drift Correction

We resolved the external feedback loop by deploying two additional modules, called $C_{q}$ and $T_{qa}$, into the system. The module $C_{q}$ computes the orientation from a given gyro value and the module $T_{qa}$ transforms the orientation to the accelerometer value that is expected from the accelerometer-assisted application.

For non-gyro signals in the measured state vector, we integrated an extra module called $T_{qn}$. The role of $T_{qn}$ is to convert the δq value, which includes elements other than the gyro error, to ΔΩ, *i.e.*, the gyro rate noise. The quaternion based indirect Kalman filter is utilized due to the linear properties of quaternion kinematics [26]. In this way, the error accumulation during the repeated transformation is significantly reduced.

The computation of the $C_{q}$ module is based on an assumption that during a time interval $\Delta t=t_{k\;+\;1}-t_{k}$, the angular velocity $\Omega =(\Omega_{x},\Omega_{y},\Omega_{z})$ is stable. Therefore, we can consider the *zero-th* order quaternion integration as a quaternion product [27] given by

(5) $$\hat{q}=\left[\begin{array}{c} \frac{\Omega }{\left|\Omega \right|}\sin\left(\frac{\left|\Omega \right|}{2}\Delta t\right)\\ \cos(\frac{\left|\Omega \right|}{2}\Delta t) \end{array}\right]⊗{\hat{q}}_{k-1}$$

The purpose of module $T_{qa}$ is to transform the orientation into the accelerometer value. $T_{qa}$ takes the quaternion $\hat{q}$ as an input and translates it into an Euler orientation. After that, the Euler orientation is translated into an accelerometer value. The details for this transformation can be found in [25].

The transformation from δΩ to ΔΩ is performed in the module $T_{qa}$. From Equation (4), we can derive $\delta q_{k+1}$ as follows

(6) $${\left[\begin{array}{c} \delta q_{1}\\ \delta q_{2}\\ \delta q_{3}\\ \delta q_{4} \end{array}\right]}_{k+1}={\left[\begin{array}{cccc} \delta q_{4} & -\delta q_{3} & \delta q_{2} & \delta q_{1}\\ \delta q_{3} & \delta q_{4} & -\delta q_{1} & \delta q_{2}\\ -\delta q_{2} & \delta q_{1} & \delta q_{4} & \delta q_{3}\\ -\delta q_{1} & -\delta q_{2} & -\delta q_{3} & \delta q_{4} \end{array}\right]}_{k}{\left[\begin{array}{c} \frac{\Delta \Omega_{x}}{\left|\Delta \Omega \right|}\sin\left(\frac{\left|\Delta \Omega \right|}{2}\Delta t\right)\\ \frac{\Delta \Omega_{y}}{\left|\Delta \Omega \right|}\sin\left(\frac{\left|\Delta \Omega \right|}{2}\Delta t\right)\\ \frac{\Delta \Omega_{z}}{\left|\Delta \Omega \right|}\sin\left(\frac{\left|\Delta \Omega \right|}{2}\Delta t\right)\\ \cos(\frac{\left|\Delta \Omega \right|}{2}\Delta t) \end{array}\right]}_{k}$$

where $\delta q_{4}$ is approximated to unity since the incremental quaternion is assumed to be a very small rotation. Calculating Equation (6), we derive

(7) $$\Delta \Omega_{x}=\frac{2\cos^{-1}\left(\gamma_{4}\right)}{\Delta t\sqrt{1-\gamma_{4}^{2}}}\gamma_{1}$$

(8) $$\Delta \Omega_{y}=\frac{2\cos^{-1}\left(\gamma_{4}\right)}{\Delta t\sqrt{1-\gamma_{4}^{2}}}\gamma_{2}$$

(9) $$\Delta \Omega_{z}=\frac{2\cos^{-1}\left(\gamma_{4}\right)}{\Delta t\sqrt{1-\gamma_{4}^{2}}}\gamma_{3}$$

where $\Delta \Omega_{x}$, $\Delta \Omega_{y}$, and $\Delta \Omega_{z}$ are the gyro rate noises on the X, Y, and Z axis, respectively. In the above equation, the *γ* value can be computed as

(10) $$\left[\begin{array}{c} \gamma_{1}\\ \gamma_{2}\\ \gamma_{3}\\ \gamma_{4} \end{array}\right]={\left[\begin{array}{cccc} \delta q_{4} & \delta q_{3} & -\delta q_{2} & \delta q_{1}\\ -\delta q_{3} & \delta q_{4} & \delta q_{1} & \delta q_{2}\\ \delta q_{2} & -\delta q_{3} & \delta q_{4} & \delta q_{3}\\ -\delta q_{1} & -\delta q_{2} & -\delta q_{3} & \delta q_{4} \end{array}\right]}_{k}^{-1}{\left[\begin{array}{c} \delta q_{1}\\ \delta q_{2}\\ \delta q_{3}\\ \delta q_{4} \end{array}\right]}_{k+1}$$

## 5. Experimental Results and Discussions

### 5.1. Preparation

**Figure 3:**
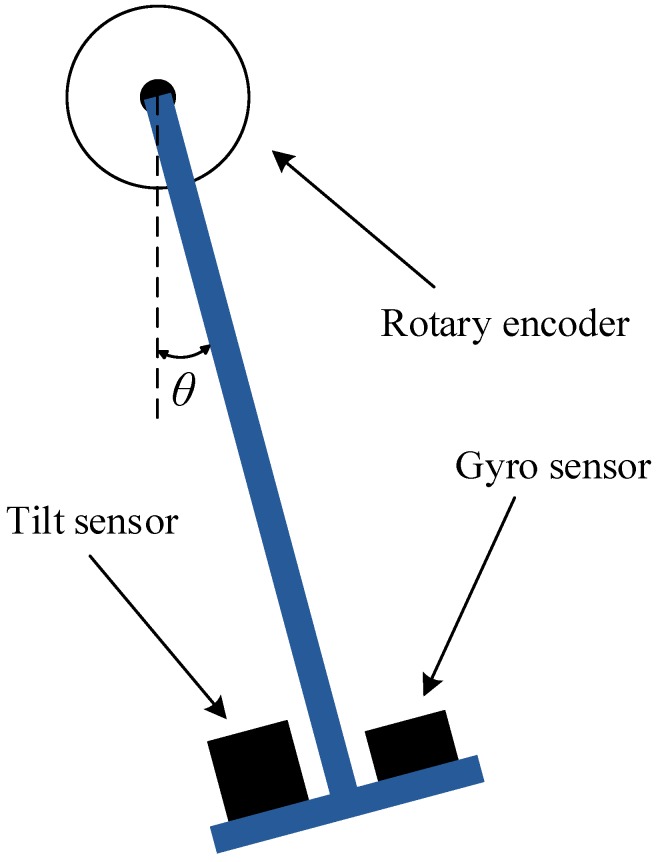
The encoder for recording the true orientation value.

In order to evaluate the performance of our proposed solution, we have conducted experiments with 41,500 samples measured by a pair consisting of a gyroscope sensor and an accelerometer sensor. The evaluations are performed based on an Android mobile device whose specifications are described in Table 1. The results are compared among the proposed approach, the legacy approach, and the reference standard provided by an encoder [28]. The true orientation values were recorded by the encoder, which can precisely capture orientations of the experimented object. A conceptual diagram of the encoder is shown in Figure 3.

### 5.2. Accuracy Evaluation

We assess the accuracy among the experimental approaches by calculating the root mean square error (RMSE) as following

(11) $$RMSE=\sqrt{\sum_{i=1}^{n}\frac{|V_{m_{i}}-V_{t_{i}}{|}^{2}}{n}}$$

where $V_{m_{i}}$ and $V_{t_{i}}$ are the value measured by the sensor and the actual value of the Euler angle at the *i-th* time, respectively, and *n* is the total number of sampled sensor data.

**Figure 4:**
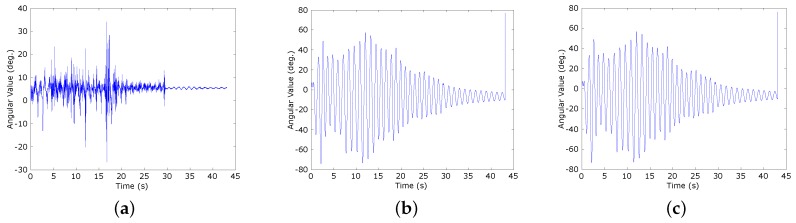
The Euler pitch angles calculated using a separate accelerometer sensor, our proposed solution, and the encoder. (**a**) Separate accelerometer sensor; (**b**) Our proposed solution; (**c**) The encoder.

Figure 4a–c show the results of Euler pitch angle calculation by using a separate accelerometer sensor, our proposed solution, and the encoder, respectively. The values and line graph shapes indicate the equivalence between our proposed solution and the encoder. However, the result of the separate accelerometer sensor is different, except the trend of the line graph. The reason is the accelerometer sensor does not respond quickly to changes of the status during high speed motion. In this Figure 4b, with our proposed solution, even though the angles are calculated by using the acceleration sensor, the result can follow the progress measured by the encoder very well without any negative effects resulting from the translational motion.

**Figure 5:**
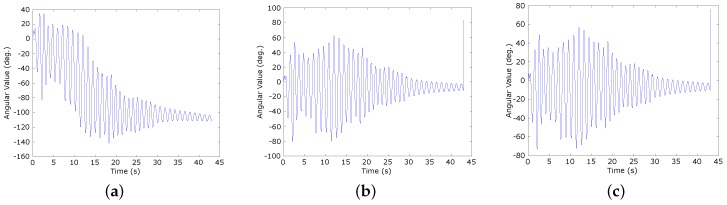
The Euler pitch angles calculated using a separate gyroscope sensor, our proposed solution, and the encoder. (**a**) Separate gyroscope sensor; (**b**) Our proposed solution; (**c**) The encoder.

Under the same analysis as applied to the accelerometer sensor, Figure 5a–c show the results of the Euler pitch angle calculation by using a separate gyroscope sensor, our proposed solution, and the encoder, respectively. Unlike the accelerometer sensor, the separate gyroscope sensor can follow the progress of angle changes. However, due to the shortcoming physical characteristics, the gyroscope sensor has a gyro drift problem that distorts the shape of the graph over time (see Figure 5a). On the other hand, with our proposed solution, the sensing information from the gyroscope sensor and accelerometer sensor is fused to compensate for each other. As a result, the output value is approximately the same as the value provided by the encoder (see Figure 5b,c).

**Figure 6:**
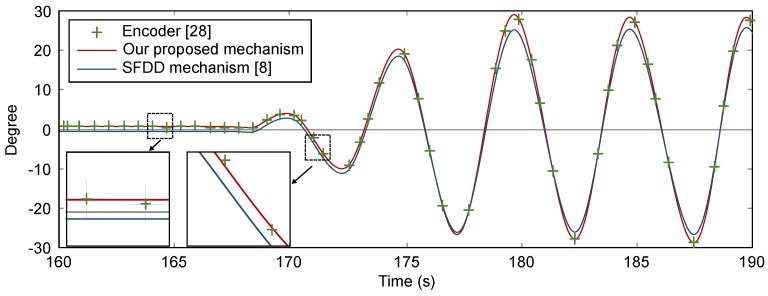
The comparison of calculated orientations among the true value, our proposed mechanism, and the sensor fusion device driver (SFDD) mechanism.

Figure 6 shows the calculated orientations comparison among the true value, our proposed mechanism, and the sensor fusion device driver (SFDD) mechanism [8]. The X-axis represents the time-lines at which the measurements were performed and the Y-axis represents the orientation values in degrees. It is observed that the proposed method provides a more accurate estimation of the orientation values than the results of the SFDD mechanism. As the recorded values are closer to each end of the range of $\left(-30^{\circ },30^{\circ }\right)$, the difference between our proposed mechanism and SFDD mechanism becomes much clear. It is noteworthy that the gyro drift is almost corrected by the proposed method. The RMSE of the SFDD mechanism and the proposed mechanism are decreased from $6.3\times 10^{-1}$ to $5.3\times 10^{-7}$, respectively.

The dependence of the calculated values on the angle range are summarized in Table 2. The calculated values are compared with the reference standard values received by the encoder.

### 5.3. Performance Evaluation

To evaluate the performance of the proposed solution, we calculated the average processing speed per sample over 4500 experimental data samples. The result shows that the proposed solution significantly increases the processing speed up to 10 times in comparison with the existing application-based methods, from 1.8579 ns to 0.18702 ns, respectively. This improvement is achieved by the movement of the fusion process from the application layer to the driver layer on the kernel level. Generally, the sample rates provided by the Android API are 4 samples/s in normal mode and 50 samples/s in fastest mode. The experiments show that the application-based method and our proposed method are able to handle 538 and 5347 samples per second, respectively.

## 6. Conclusions

In this paper, we proposed a solution to implement the sensor fusion technique at the driver layer of a mobile device. The quaternion based indirect Kalman filter and three additional modules are integrated to provide better performance and accuracy of sensing information. The external feedback loop and non-gyro error elements contained in the state vector issues are addressed before the required sensing data is delivered to the desired corresponding applications. The experimental results show that our proposed solution achieves better performance and accuracy. Moreover, any change in the device driver does not negatively affect the upper applications. The applications do not need to modify their code or be re-programmed and re-compiled to utilize the sensor fusion features. In the future research, the proposed architecture will be expanded to interact with multiple sensors and be implemented based on different devices’ hardware and operating systems.

## Figures and Tables

**Table 1:** Specifications of the experimental mobile device.

Specification	Value
CPU	S5PV210 ARM-CORTEX A8 [1 GHz]
Memory	512M DDR SDRAM
Kernel	Linux kernel 2.6.32 (Android OS)
Accelerometer sensor	3-axis accelerometer sensor
Gyroscope sensor	2-axis gyroscope sensor
Sampling frequency	100 Hz
Angle range	$\left(-90^{\circ },90^{\circ }\right)$

**Table 2:** Calculation bias of the sensing information by using difference methods.

Method	Sensor	$\left(-30^{\circ },30^{\circ }\right)$	$\left(-60^{\circ },60^{\circ }\right)$	$\left(-90^{\circ },90^{\circ }\right)$
Separate sensor	Accelerometer	22.3546	21.9589	35.5267
	Gyroscope	70.9580	72.6149	307.1894
Our proposed methods	Accelerometer	0.04875	0.0537	0.2537
	Gyroscope	1.8702	1.9309	8.6471
